# Heterogeneous alleles comprising G6PD deficiency trait in West Africa exert contrasting effects on two major clinical presentations of severe malaria

**DOI:** 10.1186/s12936-015-1045-0

**Published:** 2016-01-07

**Authors:** Shivang S. Shah, Kirk A. Rockett, Muminatou Jallow, Fatou Sisay-Joof, Kalifa A. Bojang, Margaret Pinder, Anna Jeffreys, Rachel Craik, Christina Hubbart, Thomas E. Wellems, Dominic P. Kwiatkowski, MalariaGEN Consortium

**Affiliations:** Wellcome Trust Centre for Human Genetics, University of Oxford, Oxford, UK; Medical Research Council Laboratories, Banjul, Fajara The Gambia; Wellcome Trust Sanger Institute, Hinxton, UK; Laboratory of Malaria and Vector Research, National Institute of Allergy and Infectious Diseases, National Institutes of Health, Bethesda, MD USA

**Keywords:** G6PD deficiency, malaria

## Abstract

**Background:**

Glucose-6-phosphate dehydrogenase (G6PD) deficiency exhibits considerable allelic heterogeneity which manifests with variable biochemical and clinical penetrance. It has long been thought that G6PD deficiency confers partial protection against severe malaria, however prior genetic association studies have disagreed with regard to the strength and specificity of a protective effect, which might reflect differences in the host genetic background, environmental influences, or in the specific clinical phenotypes considered.

**Methods:**

A case-control association study of severe malaria was conducted in The Gambia, a region in West Africa where there is considerable allelic heterogeneity underlying expression of G6PD deficiency trait, evaluating the three major nonsynonymous polymorphisms known to be associated with enzyme deficiency (A968G, T542A, and C202T) in a cohort of 3836 controls and 2379 severe malaria cases.

**Results:**

Each deficiency allele exhibited a similar trend toward protection against severe malaria overall (15–26 % reduced risk); however, in stratifying severe malaria to two of its constituent clinical subphenotypes, severe malarial anaemia (SMA) and cerebral malaria (CM), the three deficiency alleles exhibited trends of opposing effect, with risk conferred to SMA and protection with respect to CM. To assess the overall effect of G6PD deficiency trait, deficiency alleles found across all three loci were pooled. G6PD deficiency trait was found to be significantly associated with protection from severe malaria overall (OR 0.83 [0.75–0.92], $P = 0.0006$), but this was limited to CM (OR 0.73 [0.61–0.87], $P = 0.0005$), with a trend toward increased risk for SMA, especially in fully-deficient individuals (OR 1.43 [0.99–2.08], $P = 0.056$). Sex-stratified testing largely comported with these results, with evidence suggesting that protection by G6PD deficiency trait is conferred to both males and females, though susceptibility to SMA may be restricted to fully-deficient male hemizygotes.

**Conclusions:**

In a part of Africa where multiple alleles contribute to expression of G6PD deficiency trait, these findings clarify and extend previous work done in populations where a single variant predominates, and taken together suggest a causal role for G6PD deficiency trait itself with respect to severe malaria, with opposing effects seen on two major clinical subphenotypes.

## Background

Glucose-6-phosphate dehydrogenase (G6PD) deficiency is an X-linked enzyme disorder with a global distribution, affecting hundreds of millions of people worldwide [1]. It is a genetically heterogeneous trait, with hundreds of deficiency alleles of variable penetrance described in the literature [2], several having risen to polymorphic frequencies [3]. Prevalence of G6PD deficiency geographically correlates with the historical distribution of malaria [4], an infectious disease thought to have highly heritable differences in burden [5, 6], and a major contributor to childhood morbidity and mortality in endemic areas [7]. As such, G6PD deficiency has long been hypothesised to be a target of positive selection owing to partial protection afforded against malaria [8–11].

Genetic epidemiological evidence examining this hypothesis has been lacking in clarity, however, with past case-control association studies differing with respect to the strength and specificity of a protective effect [12–18], and even whether such an effect exists at all [19]. The difficulty in interpreting past studies lies not only in reconciling the different clinical phenotypes considered in each, but also in recognising different assumptions made in defining G6PD deficiency trait itself. With regard to the former, clinical phenotypes considered range from measures of malaria poorly correlated with disease burden, such as parasite rate or density [12, 20, 21], to more extreme phenotypes pathognomonic for severe, life-threatening malaria, including profound anaemia, respiratory distress, and cerebral manifestations such as convulsions or coma [13–15, 22]. Furthermore, World Health Organization criteria for diagnosis of severe malaria are broad, and include a wide range of symptoms and clinical findings, which vary substantially not only in terms of pathophysiology, but also with respect to prognostic value [23]. Interpreting trait carriage is also nontrivial in that G6PD deficiency is both a genetic and a biochemical trait. As a sex-linked genetic trait, hemizygous males and homozygous females fully express the deficiency trait, while heterozygous females exhibit incomplete or partial expression due to random X inactivation [24, 25]. As a biochemical trait, enzyme activity can be assayed quantitatively, but overlapping activity distributions for the different genotypic categories make qualitative assessment imperfect [16, 26, 27].

The most common G6PD deficiency allele in sub-Saharan Africa is the C202T (A-) variant, and studies of this mutation have driven most association studies in this region. However, while the C202T allele is predominant in east Africa [28], in West Africa there are several other deficiency variants (A968G, T542A, C680A) and The Gambia appears to be a notable exception to the general rule that 202T is the most prevalent allele. Recent data published by the MalariaGEN consortium has found opposing effects of the 202T allele on risk for specific severe malaria clinical subphenotypes, finding risk conferred with respect to severe malarial anaemia, and a trend toward protection with respect to cerebral malaria [29]. Given the allelic heterogeneity present in The Gambia and the robust clinical data that has been collected here over many decades [30], it represents a unique location to assess whether the effects of the 202T allele on modifying risk for specific severe malaria subphenotypes hold true for other functional alleles, and for G6PD deficiency trait as a whole, in an area where multiple alleles of variable penetrance contribute to overall prevalence of the trait [15, 31]. Presented here is a case-control association study of severe malaria and two of its major clinical presentations in The Gambia, examining all major functional alleles contributing to expression of G6PD deficiency trait in the region.

## Methods

### Ethics statement

Ethics approval was granted by The Gambia Government/Medical Research Council Joint Ethics Committee and by the Oxford Tropical Research Ethics Committee, and written informed consent was obtained from a parent or guardian prior to enrolment.

### Study cohort

The case-control cohort studied is comprised of 3836 controls and 2379 severe malaria cases, recruited at the Royal Victoria Hospital in Banjul. Malaria cases were confirmed by thin blood smear, and designated as ‘severe’ following WHO criteria, with two partially overlapping clinical syndromes identified in the majority of cases: severe malarial anaemia and cerebral malaria. Severe malarial anaemia (SMA) is defined here as packed cell volume $\le$15 % or haemoglobin $\le$5 g/dl, while cerebral malaria (CM) is defined as having a Blantyre coma score $\le$3 in the absence of other obvious causes, e.g. hypoglycaemia or meningitis. A total of 556 (23 %) of these cases met the definition for SMA and 890 (37 %) met that for CM, with 129 (5 %) cases fitting both definitions (these were excluded in the analysis of each clinical subphenotype). Control samples were derived from cord blood obtained from local perinatal clinics. A demographic summary of the cohort is provided in Table 1.

**Table 1:** Demographic summary table

	Total	(M, F)	Control	Case	(SMA, CM)	Age	(SMA, CM)
FULA	1007	534, 473	670	337	82, 116	44.7	30.9, 50.9
JOLA	938	493, 445	476	462	100, 183	52.7	33.3, 58.2
MANDINKA	1981	1070, 911	1084	897	216, 341	51.1	35.7, 55.7
OTHER	1434	689, 745	1078	356	77, 140	53.6	38.6, 60.2
WOLLOF	855	456, 399	528	327	81, 110	47.0	29.0, 54.6
TOTAL	6215	3242, 2973	3836	2379	556, 890	49.8	33.5, 55.9

Summary of cases and controls stratified by ethnicity, divided by gender, severe malaria phenotype, and mean age (months)

### Laboratory and statistical methods

Genotyping was performed on genomic DNA that was whole genome amplified via either primer extension pre-amplification or multiple displacement amplification reaction. Single nucleotide polymorphisms (SNPs) were then assayed via multiplexed genotyping on the Sequenom MASSarray platform. Automated genotype calls were manually curated via visual examination of cluster plots and mass spectra, with ambiguous genotypes re-coded as missing.

Data curation was performed using custom Perl scripts and the PLINK software package [32]. Gender designation was curated using additional X chromosome SNP data previously generated for these samples, using an inbreeding coefficient test that recodes gender based on observed heterozygosity. Any remaining heterozygous genotypes found in males were coded as missing, and individuals with ambiguous gender were excluded from the analysis. No significant frequency differences were found across genders and SNP genotype distribution in female controls did not significantly deviate from expected Hardy-Weinberg proportions ($P > 0.05$, exact test).

Association testing was conducted using custom scripts written in R [33]. A logistic regression framework was employed, using a generalised linear model assuming a binomial error distribution. In the additive (trend) model, male genotypes were coded 0/2, with females coded as 0/1/2, as suggested by Clayton [34] and in keeping with biochemical penetrance data [16, 26]. In sex-stratified tests, male hemizygotes and female heterozygotes were coded 1, with individuals lacking deficiency alleles coded as 0.

Composite G6PD deficiency trait genotype was defined as the sum of deficiency alleles present at all deficiency loci (968G + 542A + 202T) for an individual. Thus, a male was coded as 0 or 2, based on the absence or presence of a single deficiency allele across the three loci, while a female with a total of one deficiency allele present was coded as 1, and two deficiency alleles as 2 (including 13 ‘compound heterozygote’ individuals possessing a single deficiency allele at two different loci). It should be noted that this composite genotype was never greater than two because this scenario would require two deficiency alleles to co-occur on the same chromosome. Full expression of the G6PD deficiency trait, i.e. complete enzyme deficiency, was a binary genotype (0 or 1) inferred according to the absence or presence of one (male hemizygotes) or two (female homozygotes and compound heterozygotes) deficiency alleles in total across the three loci. Thus, a 202T male hemizgygote, 968G/202T female compound heterozygote, and a 542AA female homozygote would all be scored as 1 for full G6PD deficiency trait expression.

Estimated odds ratios (OR) were adjusted by including the following covariates: HbS (sickle locus) genotype, self-reported ethnicity, and sex (omitted in sex-stratified tests). All odds ratios are presented here as ‘OR [95 % CI]’.

## Results

### Association testing of *G6PD* deficiency alleles

The case-control study population included a total of 2379 Gambian children with severe malaria and 3836 cord blood control samples drawn from the same region. Genotypes were obtained at four nonsynonymous polymorphic loci within *G6PD* known to be associated with enzyme deficiency, including A968G, T542A, C680A, and C202T (Table 2), in order to comprehensively survey the genetic diversity underlying G6PD deficiency in West Africa. Three of these four SNPs were found to be polymorphic, with C680A being the lone monomorphic variant. The 968G variant was found to be the most common deficiency allele in the data set, present at a frequency of 6.6 % in controls, with 202T and 542A found at frequencies of 3.2 and 1.2 %, respectively. Each deficiency allele was first tested for association with severe malaria overall, using an additive (trend) genetic model consistent with G6PD biochemical penetrance data [16, 26], with heterozygote females treated as intermediate between non-deficient and deficient individuals, the latter being comprised of hemizygote males and homozygote females. These tests revealed a similar signal of protection for all three deficiency alleles (Table 3), suggestive of 15–26 % reduced risk, with strong evidence for the higher-frequency variant 968G (OR 0.84 [0.74–0.96], $P = 0.010$), and milder evidence for 542A (OR 0.74 [0.53–1.03], $P = 0.071$) and 202T (OR 0.85 [0.70–1.03], $P = 0.091$).

**Table 2:** Functional SNP summary table

SNP	dbSNP ID	Alias	Position	Alleles	Amino acid change	Class
A968G	rs76723693	Betica-Selma	153761240	A/G	Leu323Pro	3
C680A	rs137852328	Mexico City	153762340	C/A	Arg227Leu	3
T542A	rs5030872	Santamaria	153762655	T/A	Asp181Val	2
C202T	rs1050828	A-	153764217	C/T	Val68Met	3

For each functional SNP, the dbSNP identifier, common name, GRCh37 coordinate, major/minor alleles, amino acid residue change, and WHO biochemical classification are shown

### Stratified testing of two major clinical subphenotypes

As severe malaria encompasses several clinical syndromes that differ with respect to pathophysiology and prognostic value, stratified association testing was performed on each of the two major clinical subphenotypes predominant in The Gambia, severe malarial anaemia (SMA) and cerebral malaria (CM). Although these subphenotype tests were not equally powered to detect signals of association due to sample size differences ($N_{SMA} = 556$, $N_{CM} = 890$), they nonetheless suggested a differential direction of disease association with respect to SMA and CM for all three *G6PD* deficiency alleles (Table 3). While deficiency alleles seemed to confer protection against CM (968G: OR 0.73 [0.58–0.91]; 542A: OR 0.47 [0.23–0.99]; 202T: OR 0.82 [0.60–1.11]), they exhibited a modest, but consistent trend toward susceptibility for SMA (968G: OR 1.09 [0.88–1.35]; 542A: OR 1.36 [0.87–2.11]; 202T: OR 1.09 [0.79–1.49]). Interestingly, the 542A allele, which causes a more extreme enzyme deficiency state (WHO class 2,  1–2 % residual G6PD activity [35]), was associated with more extreme effect sizes for both subphenotypes– greater risk to SMA and greater protection from CM– than either 968G or 202T, which are of milder biochemical penetrance (WHO class 3,  10–20 % residual G6PD activity [36]).

**Table 3:** Tests of association for G6PD deficiency SNPs

	Frequency		ALL		SMA		CM	
	Control	Case (SMA, CM)	OR (95 % CI)	P value	OR (95 % CI)	P value	OR (95 % CI)	P value
	N = 3836	N = 2379 (556, 890)						
HbS	0.072	0.013 (0.010, 0.007)	0.16 (0.12–0.21)	1.1e−38	0.16 (0.09–0.29)	2.9e−09	0.11 (0.06–0.19)	8.0e−15
A968G	0.066	0.054 (0.077, 0.048)	0.84 (0.74–0.96)	0.0102	1.09 (0.88–1.35)	0.4212	0.73 (0.58–0.91)	0.0060
T542A	0.012	0.007 (0.014, 0.005)	0.74 (0.53–1.03)	0.0706	1.36 (0.87–2.11)	0.1727	0.47 (0.23–0.99)	0.0483
C202T	0.032	0.023 (0.031, 0.021)	0.85 (0.70–1.03)	0.0906	1.09 (0.79–1.49)	0.6065	0.82 (0.60–1.11)	0.1970
G6PD_trait	0.113	0.085 (0.130, 0.078)	0.83 (0.75–0.92)	0.0006	1.14 (0.96–1.36)	0.1401	0.73 (0.61–0.87)	0.0005
G6PD_trait (full)	0.063	0.050 (0.084, 0.047)	0.80 (0.63–1.01)	0.0661	1.43 (0.99–2.08)	0.0557	0.68 (0.46–1.00)	0.0524

### Association of G6PD deficiency trait with severe malaria

**Fig. 1:**
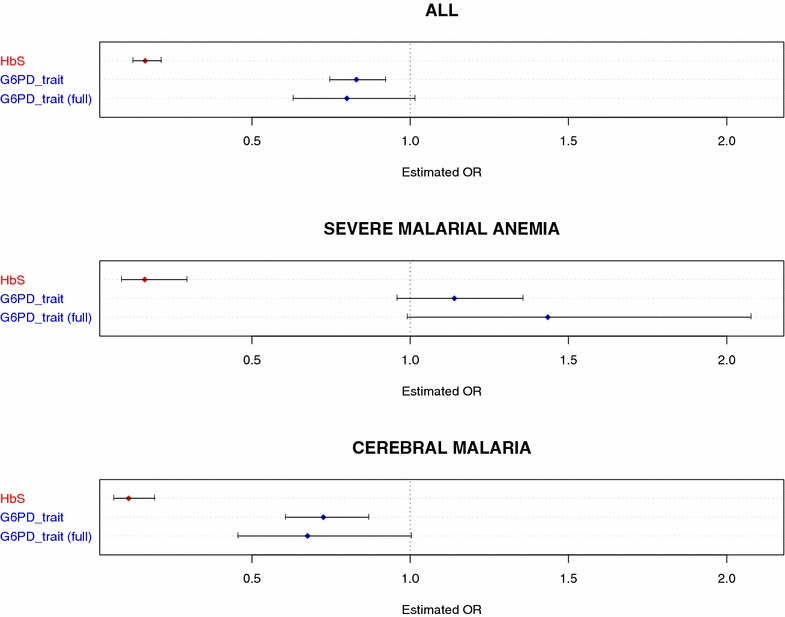
G6PD trait association tests—estimated odds ratios. Results from association tests using a composite G6PD genotype to infer G6PD deficiency trait status, where composite genotype is the total number of variant alleles present across all three deficiency SNPs (A968G, T542A, and C202T) in an individual (‘G6PD trait’). Presence or absence of full expression of the trait (i.e. full G6PD enzyme deficiency) was also tested, where presence of the ‘full’ expression of the trait required presence of one (male hemizygotes) or two (female homozygotes) deficiency alleles in total across the three loci (‘G6PD trait [full]’). Also shown are association test results for HbS (*red*). Estimated OR with 95 % CI is shown for association with severe malaria overall (ALL), as well as for each subphenotype (SMA, CM)

Since a consistent direction of association was observed with respect to SMA and CM for all three deficiency variants, and given that each contributes to the overall prevalence of G6PD deficiency trait in The Gambia, it made sense to consider them as a unit and examine the central hypothesis concerning the association of G6PD deficiency trait itself with severe malaria. Specifically, it was asked whether possession of one or more deficiency alleles at any of the three loci was associated with susceptibility to severe malaria. Thus, G6PD deficiency trait status was inferred using a composite genotype which represented the sum of deficiency alleles across all three loci, with this genotype scored in the same way as all other genotypes were, namely that hemizygous-deficient males were treated as equivalent to homozygous-deficient females, with heterozygous females being intermediate. Additionally, a binary composite genotype inferring presence or absence of full expression of the trait (i.e. full G6PD enzyme deficiency) was compiled, where presence of the trait required either one (male hemizygotes) or two (female homozygotes) deficiency alleles in total across the three loci. In these association tests (Table 3; Fig. 1), it was found that while G6PD deficiency trait exerted a strong protective effect against severe malaria overall (OR 0.83 [0.75–0.92], $P = 0.0006$), this protective effect was limited to CM (OR 0.73 [0.61–0.87], $P = 0.0005$), with a trend toward increased risk conferred to SMA, especially in fully-deficient individuals (OR 1.43 [0.99–2.08], $P = 0.056$), consistent with effects seen for the 202T deficiency allele alone in work recently published by the MalariaGEN consortium [29]. Importantly, this differential direction of association contrasts with the consistent strong protective effect across both SMA and CM clinical subphenotypes observed for sickle cell trait (Table 3; Fig. 1).

### Sex-stratified association testing

As G6PD deficiency is an X-linked trait, and the subject of gender-specificity has been conflicting in previous association studies of severe malaria [12–15], sex-stratified association tests were also conducted to examine how signals of association might vary between hemizygous males and heterozygous females. It is important to note that these tests differ with respect to: (a) statistical power, as female heterozygotes are more common than male hemizygotes, and (b) interpretation with respect to biochemical penetrance, as hemizygous males are fully-deficient, while female heterozygotes are only partially-deficient. Such caveats notwithstanding, in these tests (Table 4), evidence of protection from severe malaria overall was found for both male hemizygotes (OR 0.75 [0.58–0.96], $P = 0.023$) and female heterozygotes (OR 0.74 (0.60–0.91) $P = 0.0045$), with this protective effect confined to CM for both males (OR 0.67 [0.45–1.02], $P = 0.061$) and females (OR 0.54 [0.38–0.77] $P = 0.0006$). The trend toward susceptibility observed for SMA was only observed in fully-deficient male hemizygotes (OR 1.41 [0.96–2.08], $P = 0.083$), especially those carrying severe deficiency variant 542A (OR 2.55 [1.00–6.52] $P = 0.051$), although the lack of a similar trend in female heterozygotes is likely a reflection of their intermediate deficiency state. Interestingly, the most common G6PD deficiency allele across most of Africa, 202T, was found to be protective against severe malaria overall and the CM subphenotype only in female heterozygotes. Association testing of fully-deficient female homozygotes was limited due to low frequencies, and provided inconclusive results.

**Table 4:** Gender-stratified tests of association

Male (hemizygotes)	Frequency		ALL		SMA		CM	
	Control	Case (SMA, CM)	OR (95 % CI)	P value	OR (95 % CI)	P value	OR (95 % CI)	P value
	N = 1941	N = 1301 (309, 478)						
A968G	0.073	0.050 (0.084, 0.046)	0.70 (0.51–0.95)	0.0235	1.19 (0.72–1.95)	0.4961	0.62 (0.37–1.05)	0.0772
T542A	0.011	0.009 (0.026, 0.003)	0.84 (0.40–1.80)	0.6625	2.55 (1.00–6.52)	0.0505	0.28 (0.04–2.11)	0.2167
C202T	0.032	0.026 (0.042, 0.028)	0.87 (0.56–1.34)	0.5256	1.36 (0.68–2.72)	0.3834	0.94 (0.49–1.83)	0.8628
G6PD trait	0.118	0.087 (0.155, 0.079)	0.75 (0.58–0.96)	0.0231	1.41 (0.96–2.08)	0.0832	0.67 (0.45–1.02)	0.0608

Female (heterozygotes)	Frequency		ALL		SMA		CM	
	Control	Case (SMA, CM)	OR (95 % CI)	P value	OR (95 % CI)	P value	OR (95 % CI)	P value
	N = 1895	N = 1078 (247, 412)						
A968G	0.120	0.112 (0.148, 0.086)	0.87 (0.68–1.11)	0.2656	1.14 (0.74–1.76)	0.5654	0.63 (0.42–0.95)	0.0285
T542A	0.026	0.012 (0.022, 0.012)	0.43 (0.23–0.80)	0.0084	0.71 (0.25–2.03)	0.5244	0.41 (0.15–1.16)	0.0942
C202T	0.063	0.039 (0.048, 0.029)	0.67 (0.46–0.97)	0.0355	0.83 (0.41–1.69)	0.6065	0.49 (0.25–0.95)	0.0345
G6PD trait	0.202	0.160 (0.212, 0.124)	0.74 (0.60–0.91)	0.0045	0.99 (0.68–1.45)	0.9776	0.54 (0.38–0.77)	0.0006

## Discussion

In examining multiple G6PD deficiency alleles in The Gambia, it was found that such alleles were associated with protection from severe malaria overall, but exhibited differential effects with respect to two important clinical presentations of severe malaria, conferring protection from cerebral malaria (CM) and increasing risk for severe malarial anaemia (SMA). Taken together with prior work from populations where a single variant, C202T (A-), predominates, there is compelling evidence for a direct, causal role for G6PD deficiency trait in modification of clinical malaria subphenotypes, confirming past results demonstrating protection from CM [14, 22], as well as the more recently elucidated association with SMA [29]. Indeed, while the allelic heterogeneity present in The Gambia presents a challenge with respect to achieving adequate statistical power to detect true effects among the multiple lower frequency variants present, in analysing these variants in tandem one not only reduces type I error, but also more directly assesses G6PD deficiency trait itself independent of the genetic background upon which any particular variant lies.

As this study was limited to just one country, with its own distinct genetic background and environmental exposure history, including its own unique pattern of exposure to malaria, it will be important that further investigation be conducted in as many study populations in as diverse a range of geographic areas as possible, and there is ongoing work toward this end being conducted by the MalariaGEN consortium (manuscript in preparation). As there is substantial variation in the biochemical penetrance of G6PD deficiency alleles, it will also be useful for future work to include enzyme activity assays when feasible.

The present study provides the somewhat paradoxical result that a single gene can be associated with protection from and susceptibility to disease from the same infectious agent. This highlights the fact that severe malaria comprises distinct clinical presentations, and the extent to which these syndromes differ from one another. These findings are not unexpected, then, given that G6PD deficiency is known to predispose individuals to haemolytic anaemia in the presence of oxidant stresses from infection, drugs, and diet [37]. As the stronger signal of association is associated with cerebral malaria, the subphenotype more predictive of severe morbidity and mortality [30, 38], these findings are consistent with the malaria protection hypothesis, and suggest that further association studies at *G6PD* should distinguish between the different presentations of severe malaria.

The differential association patterns seen here also suggest possible mechanistic differences between sickle cell trait (HbS) and G6PD deficiency in the modulation of malaria clinical phenotypes.

Sickle cell homozygous and heterozygous states have been variously reported to affect invasion and growth *in vitro* [39, 40], or to decrease parasite density in natural [41–43] and experimental [41, 44] settings. Modulation of acquired immunity [45], impaired rosetting [46] or reduced cytoadherence [47] may also reduce the survival of infected HbS erythrocytes and/or mitigate processes of inflammation, and thus might explain the protection seen against both forms of severe malaria in this study.

Studies of parasite phenotypes inside the G6PD-deficient erythrocyte have been more equivocal. While some have found evidence of invasion preferences [48] or aberrant *in vitro* growth phenotypes [49], others have not [50, 51]. Similarly, evidence of decreased parasite density *in vivo* is mixed for both *Plasmodium vivax* [11, 21] and *Plasmodium falciparum* [12, 14, 20]. This raises the possibility that the protection afforded by G6PD deficiency against cerebral malaria might depend on genetic background or operate through some mechanism other than parasite survival inside the enzyme-deficient red cell. Given that G6PD is an important housekeeping enzyme with a wide distribution in the body, it is interesting to consider, for example, how its role in endothelial function (via nitric oxide synthase [52, 53]), or in the respiratory burst of immune cells (via NADPH oxidase [54]), might contribute to malaria protection.

Importantly, differences in the protection afforded by HbS and G6PD deficiency highlight the need to study clinical biomarkers and conduct *in vitro* experiments relevant to severe manifestations of malaria. Ultimately, if knowledge gained from investigations surrounding the malaria protection hypothesis is to inform clinical treatment, a better understanding of the molecular mechanisms as they relate to severe disease will be crucial.

## Conclusions

In a region of West Africa with significant functional allelic heterogeneity, these data confirm that G6PD deficiency state is protective against severe malaria, but in contrast to sickle cell trait, this protection is limited to the CM subphenotype, with possible increased risk conferred to SMA. These results support the malaria protection hypothesis, but also suggest important mechanistic differences between the malaria-protective mechanisms of G6PD deficiency versus sickle cell trait.

## Abbreviations

G6PD: glucose-6-phosphate dehydrogenase
SMA: severe malarial anaemia
CM: cerebral malaria
WHO: World Health Organisation
SNP: single nucleotide polymorphism
HbS: sickle cell trait
OR: odds ratio
CI: confidence interval
